# Metabolic Syndrome in First Episode Schizophrenia, Based on the National Mental Health Registry of Schizophrenia (NMHR) in a General Hospital in Malaysia: A 10-Year Retrospective Cohort Study

**DOI:** 10.3390/ijerph15050933

**Published:** 2018-05-07

**Authors:** Albert Muh Haur Lee, Chong Guan Ng, Ong Hui Koh, Jesjeet Singh Gill, Salina Abdul Aziz

**Affiliations:** 1 Department of Psychiatry, Hospital Kuala Lumpur, 50586 Kuala Lumpur, Malaysia; albert_imu@hotmail.com (A.M.H.L.); salinaziz@gmail.com (S.A.A.); 2 Department of Psychological Medicine, Faculty of Medicine, University of Malaya,50603 Kuala Lumpur, Malaysia; Malaysia; ohkoh@um.edu.my (O.H.K.); jesjeet@um.edu.my (J.S.G.)

**Keywords:** metabolic syndrome, schizophrenia, risk

## Abstract

Schizophrenia has been linked with various medical comorbidities, particularly metabolic syndrome. The number of studies on this aspect is lacking in Malaysia. (1) Objective: To investigate metabolic syndrome rates and its associated factors. (2) Method: This is the first 10-year retrospective-outcome study of patients with first episode schizophrenia in Malaysia. Out of 394 patients diagnosed with first episode schizophrenia and registered with the National Mental Health Registry of Schizophrenia (NMHR) in the General Hospital Kuala Lumpur (GHKL) in 2004–2005, 174 patients consented to participate in the study. They were interviewed using a Schizophrenia outcome questionnaire and the International Physical Activity Questionnaire (IPAQ). The diagnosis of metabolic syndrome was made using the National Cholesterol Education Program—Third Adult Treatment Panel (NCEP ATP III). (3) Results: All patients’ weight, body mass index, fasting blood sugar, and blood pressure are significantly increased. Sixty-three subjects (36.2%) developed metabolic syndrome while 36 (23.2%) were hypertensive, and 41 (28.1%) were diabetic. Use of fluphenthixol depot (CI = 1.05–5.09, OR: 0.84, *p* = 0.039), reduced physical activity (CI = 0.13–1.00, OR: −1.04, *p* = 0.049), and substance use disorder (CI = 1.40, 13.89, OR: 1.48, *p* = 0.012) were significantly associated with metabolic syndrome based on univariate analysis. In further multivariate analysis, comorbid substance abuse was the only significant factor associated with metabolic syndrome after adjusting for physical activity and intramuscular depot. (4) Conclusion: Patients with schizophrenia are at high risk of metabolic syndrome. It is important to address substance use problems as an important risk factor of this comorbidity.

## 1. Introduction

Schizophrenia is a major mental disorder that alters the patient’s perception, thought, affect, and behavior. According to the World Health Organization (WHO), it is one of the major mental illnesses that leads to the global burden of disease [1], as the 14th most moderate and severe disability and 6th in the list for the most causes of years lost due to disability (YLD) [1]. There is research reporting that patients with schizophrenia have a shorter life expectancy, as much as 6–7 years shorter [2]. Allebeck et al. [3] examined the overall mortality among the cohort and found that after excluding for suicide, the mortality rate is twice as high as the general population among patients with schizophrenia. Hennekens CH et al. [4] reported that schizophrenia patients have 20% less life expectancy, mainly due to coronary heart disease (CHD) [5], which is strongly related to metabolic syndrome. Patients with schizophrenia were also reported to have a 2–3-fold increased risk of dying from cardiovascular events [6]. From a review by Marc De Hert, patients with schizophrenia have a 2–3 times higher rate of developing metabolic syndrome, while from the Clinical Antipsychotic Trials of Intervention Effectiveness (CATIE) study, approximately 30% of schizophrenia have metabolic syndrome [7].

Metabolic syndrome consists of obesity, elevated blood pressure (i.e., hypertension (HPT)), impaired insulin sensitivity, and dyslipidemia. The International Diabetic Federation consensus report (2006) estimates as high as 20%–25% of the world’s adult population has metabolic syndrome and patients with schizophrenia are at even higher risk to develop such a syndrome. While diet intake and less active physical activity are to blame, treatment, especially atypical antipsychotics, also contributes to the syndrome. In particular, medications such as olanzapine is known to promote insulin resistance and weight gain. Over the past 20 years, there have been more concerns about metabolic syndrome because of the risk of developing Type 2 Diabetes Mellitus (DM) and coronary heart disease [8,9,10], which are major causes of morbidity and mortality. First episode patients may develop metabolic problems after initiation of antipsychotics, especially the atypical ones, but since they are drug naïve, not much is yet known about the reasons why they develop metabolic conditions. One should, however, suspect similar profiles as chronic patients, making them equally susceptible.

Schizophrenia patients are known to have poorer health compared to the general population. In a selective review [11], the most observed cause of mortality in patients with schizophrenica is cardiovascular events. These populations are also associated with higher rates of obesity, metabolic aberrations, substance use (smoking, alcohol), lack of exercise, and poor diet. [11] These risk factors might put them at a higher risk of developing medical illnesses.

In addition, patients with schizophrenia are prone to health issues, especially metabolic syndrome secondary to its treatment (antipsychotics). Antipsychotic medications are known to lead to significant weight gain and increase adiposity-independent changes in insulin sensitivity and lipid metabolism [12].

In Malaysia, the metabolic syndrome issue is equally worrisome. Wan Nazaimoon Wan Mohamed et al. (2010) revealed that the prevalence of metabolic syndrome in Malaysian adults using WHO, Third Adult Treatment Panel (ATP III), and International Diabetes Federation (IDF) definitions was 32.1%, 34.3%, and 37.1%, respectively [5].

Malaysia has a high prevalence of metabolic syndrome at 34.3% (2011) [13] compared with other countries in Asia, e.g., China (9.8%), India (24.9%), Korea (13.1%), Hong Kong (13.4%), Taiwan (16.4%), and Thailand (15%) [6,14,15,16,17,18,19]. Research done by Paul Nestel MD. et al. (2007), using Asian-adapted definitions of obesity (Body mass index (BMI) ≥ 25 kg/m^2^) and increased waist circumference (for male ≥90 cm; for female ≥80 cm), reported that the prevalence of metabolic syndrome in East and Southeast Asia appears to be between 10% and 30% only [17]. As per the literature reviewed, the prevalence of metabolic syndrome among schizophrenia patients is higher than the general population in most of the Asian countries. For example, Japan recorded a prevalence of 27.5% [20], while India registered 33.3%, compared to 11.9% among the general population [21]. Thailand reported a prevalence of 22.8%, compared to 15% of the general population [22]. Despite expecting a higher prevalence of metabolic syndrome among schizophrenia patients, the data is not known among patients with schizophrenia 10 years after initial diagnosis. Up till now, there is no long-term outcome study of metabolic syndrome schizophrenia patients in Malaysia.

The aim of the study is to determine the rate of metabolic syndrome and its associated factors among patients with the first diagnosis of schizophrenia 10 years prospectively in Malaysia.

## 2. Materials and Methods

This is a retrospective 10-year cohort study to determine the metabolic outcome and its associated factors among patients with first episode schizophrenia in Malaysia.

All newly diagnosed schizophrenia patients who came to Hospital Kuala Lumpur and registered with the National Mental Registry (NMHR) from 1 January 2004 to 31 December 2005 were identified. Those who had defaulted treatment were called and rescheduled to see investigators. They then underwent a face-to-face interview. Those who had lost contact or follow up were identified as well.

### 2.1. Inclusion Criteria

1. Diagnosed with schizophrenia based on DSM IV-TR.
2. First episode contacts and registered with the national registry (NMHR) in 2004–2005.
3. Adult age between 18 and 60 years of age.
4. Consent either from patients or family members/caretakers.

### 2.2. Subjects Who Were Excluded from the Study

1. Patients who had passed away (confirmed with National Registry Department).
2. Change of diagnosis since 2004/2005.
3. Lost to follow up.

All the parameters for metabolic syndrome were measured and the latest blood investigations taken were traced.

NCEP ATP III Criteria (National Cholesterol Education Program—Third Adult Treatment Panel) (Grundy et al. 2004) were selected with adjustment for waist size in Asian subjects (World Health Organization, 2000). These include at least three of the following criteria:

(a) Central obesity (male ≥90 mm, female ≥80 mm)
(b) High triglyceride (≥1.7 mmol/L)
(c) A low HDL cholesterol concentration (<1.3 mmol/L)
(d) Elevated blood pressure (SBP ≥ 130 mmHg; SBP ≥ 85 mmHg)
(e) Glucose intolerance (fasting blood glucose ≥ 6.1 mmol/L)

### 2.3. Instruments Used

(a) Schizophrenia outcome study questionnaire

This is a self-generated questionnaire to record patients’ demographic, physical, and clinical data. The assessment also includes the Personal and Social Performance scale.

(b) International physical activity questionnaire (IPAQ)

IPAQ was developed during a conference in Geneva in 1998 to measure health-related physical activity in general populations, which includes time spent in vigorous intensity activity, moderate intensity activity, and walking, which lasted at least 10 min or more per session. It is suitable for use in regional, national, and international monitoring and survey systems and for research projects and public health program planning and evaluation. 

IPAQ has been extensively tested for its reliability and validity around the world and translated into many languages including Malay.

It was designed and tested for populations aged 15–69 years. Specific activities within each major heading with its intensity is defined as the ratio of work metabolic rate to a standard resting metabolic rate (MET). Energy expenditure in MET-minutes, MET-hours, kcal, or kcal per kilogram body weight can be estimated for specific activities by type or MET intensity. MET is an easy way to count the energy cost of different types of physical activities as a multiple of the resting metabolic rate. 

### 2.4. Formulation

MET—multiple of the resting metabolic rate

MET-min—multiplying the MET score of an activity by the minutes performed.

MET-minute score is equivalent to kilocalories for a 60 kg person.

Therefore kilocalories = MET-min X (Weight of body in kilograms/60 kg).

The score from METs can then be divided into three groups: low, moderate, or high.

### 2.5. Statistical Analysis

Data collected were analyzed using version 23 of Statistical Package for Social Science (SPSS) (IBM, Armonk, NY, USA). Descriptive analysis was done for the baseline characteristic and clinical features. Simple logistic regression was used to analyze the association between sociodemographic and clinical variables with metabolic syndrome. Significant variables were included into multiple logistic regressions to examine the influence of independent variables with metabolic syndrome and its parameters. All analysis was 2-tailed with alpha value of 0.05.

## 3. Results

Out of the 394 patients registered in 2004–2005, there were 37 patients who had passed away (cross checked with the National Registry) and 117 of them were lost to follow up and not traceable in the record. This is either because they have moved to a new place or had changed their contact number. Fifty-eight subjects were not able to be assessed, as they were not able traceable in the system (due to wrong entry of name or identity number).

The mean age of the group was 42.3 years old (SD = 10.7) and mostly male (64.9%). The majority of them were single (70.1%), while 11.5% of them were divorced, separated, or widowed. Most of the subjects had at least secondary education (69%) and 32 of them (18.4%) completed tertiary school. Among the subjects, only 36.2% were employed. From the cohort, 30.5% of the subjects had a family history of diabetes mellitus, while 43.1% of them had a family history of hypertension (Table 1).

### Clinical Data

Comorbid substance use among patients with mental illness is high, especially among those with schizophrenia. Of the subjects in this study, 15.5% had comorbidity of substance abuse, mainly misuse of cannabis (44.4%) and methamphetamine (63%). There were nine of them (5.2%) who suffered from depression (Table 2).

After 10 years of treatment and follow up, 22 of them (12.6%) were diagnosed to have diabetes mellitus, 23 of them (13.2%) had hypertension, while 19 (10.9%) of them had dyslipidaemia and 4 (2.3%) had Ischemic Heart Disease (IHD). A total of 102 (58.6%) subjects were on atypical antipsychotic and 62 of them (35.6%) on typical antipsychotic. There was a total of 67 (38.5%) subjects on depot injection, with I.M. Fluanxol the most common among all (73%) (Table 2).

Table 3 shows the study subjects’ metabolic parameters at baseline and 10 years later. At baseline, the mean (SD) weight of the study subjects was 58.6 kg (SD = 12.28) with the mean BMI of 22.38 kg/m^2^ (SD = 4.15); 102 (58.6%) of the subjects had normal BMI, while 22 (12.6%) of them were overweight and 37 (21.3%) were obese. The mean systolic blood pressure (SBP) was 117.4 (SD = 14.09) while mean diastolic blood pressure (DBP) was 73.8 mmHg (SD = 9.82). The median Interquartile range (IQR) of fasting blood sugar (FBS) was 4.4 mmol/L (SD = 1.0). Among all subjects at baseline, 6 (3.8%) patients were hypertensive and 7 of them had diabetes (4.4%). There was no measurement of the waist circumference and fasting lipid profile at the baseline. As such, the rate of metabolic syndrome at baseline cannot be determined.

After 10 years, at the current assessment, all the metabolic parameters were significantly increased (Table 3). The mean weight was 70.1 kg (SD = 17.12), while the mean BMI had increased to 26.4 kg/m^2^ (SD = 5.76). Out of all subjects studied, only 27.6% of subjects were within normal BMI, while 24 (16.6%) patients were overweight and 81 (55.9%) patients had fallen into the obese categories (Table 3).

For the waist circumference, the mean was 88.41 cm (SD = 12.93), 43 of the male subjects (45.7%) had obese waists, and 51 (54.3%) of them had normal waists compared with the females with 18 (31%) of them having normal waists and 40 (69%) of them with obese waists. There was a significant difference between the females who had larger waist circumference compared with the males.

After 10 years, 63 (36.2%) patients developed metabolic syndrome, with 23.2% of them being hypertensive and 41 (28.1%) of them having diabetes. (Table 3) For the other metabolic parameters, the mean for the total cholesterol was 5.04 mmol/L (SD = 1.18) and LDL was 3.01 mmol/L (SD = 1.06), while the median (IQR) for HDL is 1.2 mmol/L (SD = 0.60) and TG is 1.2 mmol/L (SD = 1.1). Forty-nine of them (23.9%) had high LDL, 88 (42.9%) of them had low HDL, and another 49 of them (35.8%) had high TG levels. (Table 3).

Table 3 shows that there were significant differences between all the metabolic parameters at baseline and after 10 years among the schizophrenic patients, namely, the systolic BP, diastolic BP, BMI, and fasting blood sugar level (*p* < 0.001).

In univariate analysis, the results showed that depot antipsychotic injection was associated with metabolic syndrome, and in particular, fluanxol, (OR = 2.31, CI = 1.05–5.09). The moderate to high physical activity group was significantly associated with lower metabolic syndrome (OR = 0.36, CI = 0.13–1.00). Another significant finding was the comorbidity of substance abuse/substance use disorder (OR = 1.48, CI = 1.40–13.89 (Table 4).

Three significant variables associated with metabolic syndrome, i.e., physical activity, I.M. Fluanxol, and comorbid substance abuse, were included into the multiple regression analysis. The results of the multiple logistic regression showed that substance abuse was the only significant factor associated with metabolic syndrome (Table 5). 

## 4. Discussions

Schizophrenia patients are well known to have higher morbidity and mortality compared with the general population. Some literatures suggest that they might have a shorter life expectancy of 20% less than others [23]. Among all, metabolic syndrome is one of the major leading causes of morbidity and mortality among patients with schizophrenia, as it leads to a higher risk of coronary heart disease and myocardial infarction [24,25]. Some researchers have argued that those with mental illness are lacking in physical activity and have poor nutritional intake, a sedentary life-style, increased rates of smoking, and suffer from abnormalities of the hypothalamic–pituitary–adrenal axis [10,26,27,28,29]. In the current study, we included all of the patients registered with the National Mental Health Registry (NMHR) of Schizophrenia at Hospital Kuala Lumpur in 2004–2005. There was a cohort of 394 subjects, of whom there were more males. The ethnicity of the group was the same as the Malaysian population, which consists mainly of Malays (51.7%), Chinese (33.9%), and Indians (10.9%). Most of them were single and had an educational level up to secondary school. According to the NMHR outcome study published in 2008 [30], 70% were never employed or unemployed at the time of registration. After 10 years, 63.8% remained unemployed. The median of duration of untreated psychosis (DUP) was 12 months. Schizophrenia patients have high risk of comorbidities. In a study by Peter F. Buckley [31], he found that anxiety disorder (15%–23%), substance abuse (47%), and depression (50%) were among the most common comorbidities in schizophrenia. A review by Raphael J. Braga (2013) also found that anxiety disorders were the most common comorbidity in schizophrenia [32]. In the current research, we found that 15.5% of the study subjects were involved in substance use, mainly methamphetamine (63%) and cannabis (44.4%). Other substances involved were opiates and alcohol. There were 9 subjects (5.2%) diagnosed that with comorbid depression. Depression is common in schizophrenia but is often underestimated or under-reported. This may be due to the overlapping of depressive symptoms and the negative symptoms of schizophrenia, as well as the extrapyramidal side effects of the medications [33,34]. Literatures have reported that up to 30%–40% of patients with schizophrenia having depressive symptoms during follow up post-discharge [31]. Schizophrenia with depression is associated with poorer outcome due to work impairment, low activity, and suicidal tendencies. Previous literature also reported that 20% of the female patients who were suffering from schizophrenia had depression [35]. Some even reported that the rates might be as high as 20%–80% [26]. 

The prevalence of metabolic syndrome found in the current research was lower than previous reports in the country [5]. This could mainly be due to the differences in the study design. The current study is a retrospective cohort study on patients newly diagnosed with schizophrenia 10 years ago. There were a large number of subjects that passed away or were lost to follow up in the current study. The causes of death or their risk of metabolic syndrome were unknown. In contrast, other studies were mostly cross-sectional studies. In the cross-sectional studies, both chronic and new cases of schizophrenia were included. The measurement of metabolic syndrome was done at the point of study. Furthermore, the current findings could be underestimated and there were no standardized instruments used while making the comorbid diagnoses in a study conducted in the United States. Despite high awareness of the presence of depression among patients with schizophrenia, more than a quarter of psychiatrists hardly ever or even never prescribe adjunctive antidepressants [34].

We found that there was a worsening of most metabolic parameters in our cohort. The mean weight at the initiation of the study was 58.6 kg (SD = 12.3) and mean BMI was 22.38 (SD: 4.2), which increased to 70.1 kg for the mean weight and 26.35 for the mean BMI. At the current measurement after 10 years of schizophrenia, 45.7% of male patients had obese waist circumferences while for 69% of females had waist circumferences of more than 80 cm. There were significantly more females than males with obese waists. For fasting blood glucose, the median of blood glucose was 4.4 mmol/L at baseline and increased to 5.0 mmol/L after 10 years.

In the current study, we found that 36.2% of the study subjects had metabolic syndrome after 10 years of schizophrenia diagnosis. Lack of physical activities, use of depot antipsychotic injection, and illicit substance use were the significant associated factors for metabolic syndrome. As reported in previous studies, the life-style factor is strongly associated with development of metabolic syndrome. The most prominent factor would be physical activity. David E. Laaksonen et al. (2002) [36] found that low levels physical activity can predict the chance of developing metabolic syndrome. With an increase in physical activity, the risk can be reduced as much as 75% [36]. One study also described vigorous and moderate physical activity to have odd ratios of 0.52 (95% CI: 0.40, 0.67) and 0.78 (95% CI: 0.63, 0.96), adjusted for the confounders [37].

Antipsychotics are commonly associated with metabolic syndrome. Not only that, some studies found antipsychotic use is associated with increased cardiovascular heart disease (CHD), hence increased morbidity and mortality among patients with schizophrenia [38,39,40]. In other words, antipsychotic use not only increased the risk of metabolic syndrome, but increased CHD risk as well. Surprisingly, atypical antipsychotics were commonly not shown to be a significant factor in the current study. This could be explained by the financial cost of using atypical antisychotics in Malaysia. The only atypical antipsychotic that was subsidized and commonly prescribed in the current study setting was risperidone. It was not only used as a first line but as part of a combination treatment for schizophrenia. Risperidone has a relatively lower risk of metabolic syndrome as compared to other atypical antipsychotics like olanzapine and clozapine [41]. The duration and dosage of the atypical antipsychotics could be lower than optimal, although they were not collected in the current study.

Substance abuse has been a major issue among schizophrenic patients. Schizophrenic patients with substance use issues are known to have poorer outcomes and compliance [40,42]. In addition, those with substance problems tend to have more psychotic symptoms, agitation, and aggression. They also had less support, with a higher risk of blood borne infection (e.g., needle sharing) and also used medical facilities more frequently [40]. They had more frequent relapses and admissions to hospital [43]. Another study showed that prolonged use or dependence on substances was one of the predictors of developing metabolic syndrome [44].

In the literature looking into relationships between substance abuse/dependence and metabolic syndrome, Vermani et al. 2007 proposed a few theories that leads to metabolic syndrome among drug abuse [45]. These substance users were noncompliant to treatment of diabetes, leading to higher rates of diabetic complications. Nutritional education, i.e., eating better and healthier, was found to improve the outcome in substance abusers [46]. The authors also argued that cognitive deficits among schizophrenics with substance use, such as methamphetamine abuse, might play a role in the development of metabolic abnormalities or nutritional problems [45]. Furthermore, oral health issues in methamphetamine abusers [46,47] results in poor oral hygiene, poor chewing mechanism, xerostomia, more rampant caries, and excessive tooth wear, all leading to poorer digestion and hence nutrition absorption and subsequently may lead to metabolic syndrome. Virmani et al. also found proposed that substance dependence might lead to cell damage and excitotoxicity. In addition, energy producing abnormalities lowering the antioxidant potential of the cells might be the pathogenesis of metabolic syndrome among substance abusers.

According to Zorrick T et al., methamphetamine withdrawal can be divided into an acute phase (7–10 days) and a subacute phase (which can last 2–3 weeks). During the acute withdrawal stage, abusers will have an increase in appetite and hyperphagia on top of a craving for methamphetamine [47]. The user will have mood symptoms, especially depressive symptoms. To overcome the depressed mood, they will crave for “happy” food, which is high in glucose and carbohydrates, therefore indirectly leading to metabolic syndrome.

Evidence also showed that long-term use of methamphetamine leads to changes in both α2-adrenoceptor and β-adrenoceptor, which are responsible for homeostasis of food intake. That is, 24 h mean β-adrenoceptor (which acts on lateral hypothalamus) binding is reduced and α2-adrenoceptor (which binds to medial hypothalamus) binding is increased upon methamphetamine withdrawal, which explains the feeding patterns during withdrawal, i.e., rebound feeding occurs [48]. Schizophrenia patients with comorbid substance abuse issues also tend to neglect their health. They might care less about their physical health, eat less healthy, smoke, and neglect self-health. This leads to a high risk of developing physical illnesses, for example, dyslipidaemia and diabetes, which contributes to the occurrence of metabolic syndrome [16].

This was the first long-term outcome study (10 years) on metabolic syndrome in schizophrenia conducted in the country. The study was conducted at one of the biggest general hospitals in the country. The subjects represent the population in the capital of the country, Kuala Lumpur. This provides a good catchment area, which represents the general population of the city.

There were some limitations in the current study. There are many factors that may lead to metabolic syndrome. One of them is dietary intake, which was not looked into in the current study. The drop out/missing data/loss-to-follow-up rate was relatively high. Although the author failed to trace many subjects for whom no record or contact available, there was a possibility of recall bias. The IPAQ questionnaire examines the physical activity for the past one week. That does not guarantee the person has the same amount of physical activity before this period. Therefore, research might not represent the true picture of the cohort. The cohort might be under-reporting their physical activity as well. Another limitation is that some of the metabolic parameters were not measured at the baseline. For example, blood results and waist circumference variables are not available at baseline. This was because when the registry was started, metabolic syndrome was not the main outcome emphasized. Physical activity was not measured. The sample size for the substance abuser group was relatively small. The authors assumed those with comorbid substance use might be lost to follow up. Due to the small subject pool, the author was unable to subanalyze the association of each individual substance and metabolic syndrome.

## 5. Conclusions

Metabolic syndrome is on the rise more so in patients with Schizophrenia. The current study found that 36.2% of schizophrenia patients had metabolic syndrome after 10 years. The rate is much higher compared with other countries in Asia. Substance abuse is an important factor that is associated with metabolic syndrome in schizophrenia beside physical activity and intramuscular flupethixol depot injection. Reducing the risk of metabolic syndrome in these groups of schizophrenic patients is important.

## Figures and Tables

**Table 1:** Sociodemographic characteristics of the study participants.

Variables	Subtypes	Mean, n (%)	Mean, (SD)
Age			42.3 (10.67)
Gender	Male	113(64.9)	
	Female	61 (35.1)	
Ethnicity	Malay	90 (51.7)	
	Chinese	59 (33.9)	
	Indian	19 (10.9)	
	Others	6 (2.9)	
Religion	Islam	94 (54.0)	
	Buddhist	42 (24.1)	
	Hinduism	12 (6.9)	
	Others	26 (14.9)	
Marital Status	Single	122 (70.1)	
	Married	32 (18.4)	
	Others	20 (11.5)	
Education Level	No School	5 (2.9)	
	Primary School	17 (9.7)	
	Secondary School	120 (69.0)	
	Tertiary School	32 (18.4)	
Employment	Unemployed	111 (63.8)	
	Employed	63 (36.2)	
Family History of DM	Yes	53 (30.5)	
	No	101 (58.0)	
	Unknown	20 (11.5)	
Family History of HPT	Yes	75 (43.1)	
	No	79 (45.4)	
	Unknown	20 (11.5)	

HPT = Hypertension.

**Table 2:** Clinical characteristics of the study subjects.

Variables	Subtypes	n (%)
Comorbidity:		
None		130 (74.7)
Substance		27 (15.5)
	Cannabis	12 (44.4)
	Opiates	5 (18.5)
	Methamphetamine	17 (63.0)
	Inhalants	1 (3.7)
	Alcohol	4 (14.8)
	Others	2 (7.4)
Depression		9 (5.2)
Medical Illness		
	Diabetes Mellitus	
	Yes	22 (12.6)
	No	137 (78.7)
	Unknown	15 (8.6)
	Hypertension	
	Yes	23 (13.2)
	No	136 (78.2)
	Unknown	15 (8.6)
	Ishaemic heat disease	
	Yes	4 (2.3)
	No	155 (89.1)
	Unknown	15 (8.6)
	Dyslipidaemia	
	Yes	19 (10.9)
	No	140 (80.5)
	Unknown	15 (8.6)
Smoking	Yes	97 (55.7%)
	No	77 (44.3%)
Treatment		
Antipsychotic Oral		n (%)
Typical	Total	62 (35.6)
	Chlorpromazine	11 (17.4)
	Haloperidol	14 (22.6)
	Trifluoperazine	2 (3.2)
	Perphenazine	1 (1.6)
	Sulpiride	28 (45.2)
	Others	6 (9.8)
Atypical	Total	102 (58.6)
	Risperidone	57 (55.9)
	Olanzepine	21 (20.6)
	Quetiapine	8 (7.8)
	Clozapine	11 (10.8)
	Aripiprazole	3 (2.9)
	Others	2 (2.0)
Depot	Total	67 (38.5)
	Modecate	11 (16.4)
	Fluanxol	49 (73.1)
	Zuclopenthixol	2 (3.0)
	Paliperidone	4 (6.0)
	Others	1 (1.5)
Combination therapy	Yes	62 (35.6)
Type of Combination	Risperidone and I.M. Fluanxol	24 (39.3)
	Risperidone and I.M. Modecate	3 (4.9)
	Chlorpromazine and I.M. Fluanxol	5 (8.2)
	Sulpiride and I.M. Fluanxol	4 (6.6)
	Other combinations	25 (41.0)

I.M. = Intramuscular injection.

**Table 3:** Metabolic parameter measurements at baseline and after 10 years.

Variables	After 10 Years	Baseline	*p* Value
Weight (kg), mean (SD)	70.05 (17.12)	58.6 (12.28)	-
Height (cm), mean (SD)	162.79 (8.69)	161.7 (8.59)	<0.01
BMI, mean (SD)	26.35 (5.76)	22.38 (4.15)	<0.01 *
Normal, n (%)	40 (27.6%)	102 (58.6)	
Overweight, n (%)	24 (16.6%)	22 (12.6)	
Obese, n (%)	81 (55.9%)	37 (21.3)	
Waist (cm), mean (SD)	88.41 (12.93)		-
Waist by Gender:			-
*Male:*			
Obese waist, n (%)	43 (45.7)		
No Obese waist, n (%)	51 (54.3)		
*Female:*			
Obese waist, n (%)	40 (69.0)	-	
No Obese waist, n (%)	18 (31.0)	-	
Total cholesterol, mean (SD)	5.04 (1.18)	-	-
LDL, mean (SD)	3.01 (1.06)	-	-
Normal level, n (%)	88 (42.9)		
High level, n (%)	49 (23.9)		
HDL, median (IQR)	1.2 (0.6)	-	-
Normal HDL	49 (35.8)		
Low HDL	88 (64.2)		
TG, median (IQR)	1.2 (1.1)	-	-
Normal Level	88 (64.2)		
High Level	49 (35.8)		
FBS, median (IQR)	5 (1.1)	4.4 (1.0)	<0.01 **
Normal	105 (71.9)	128 (80.5)	
Impaired	16 (11.0)	9 (5.7)	
DM	25 (17.1)	7 (4.4)	
Systolic BP (mmHg), mean (SD)	128.39 (12.87)	117.4 (14.09)	<0.01 *
Diastolic BP (mmHg), mean (SD)	80.94 (9.21)	73.8 (9.82)	<0.01 *
Metabolic syndrome, n (%)	63 (36.2)		-

Note: Table 3 No measurement of the waist circumference and fasting lipid profile at the baseline. * Paired *t*-test, ** Wilcoxon Signed Rank Test.

**Table 4:** Association factors of metabolic syndrome in patients with schizophrenia.

Variable		B	Unadjusted Odd Ratio	95% CI Low Up		*p* Value
5.1 Smoking						
	Yes	0.379	1.46	0.73	2.91	0.283
5.2 Age:	<43 year					
	>43 year	−0.147	0.86			0.392
5.3 Education:	No/1′ edu	0	1			
	2′/3′ edu	−1.266	0.28	0.08	1.06	0.061
5.4 Gender:						
Male	No	0	1			
	Yes	0.18	1.20	0.60	2.39	0.610
5.5 Ethnicity:						
Malay	No	0	1			
	Yes	−0.545	0.58	0.29	1.15	0.117
Chinese	No	0	1			
	Yes	0.365	1.44	0.70	2.95	0.317
Indian	No	0	1			
	Yes	0.610	1.84	0.59	5.71	0.290
5.6 Medication						
Typical	No					
	Yes	0.464	1.59	0.76	3.35	0.221
Atypical	No					
	Yes	−0.696	0.50	0.24	1.04	0.064
5.7 Depot	No					
	Yes	0.812	2.25	1.10	4.60	0.026
5.7.1 Modecate	No					
	Yes	0.735	2.09	0.52	8.44	0.302
Fluanxol	No					
	Yes	0.835	2.31	1.05	5.09	0.039
5.8 Substance	No					
	Yes	1.482	4.40	1.40	13.89	0.012
5.9 Depression	No					
	Yes	−0.174	0.841	0.20	3.51	0.812
6.0 Physical Activity	No					
	Yes	−1.036	0.355	0.126	1.00	0.049
6.1 Duration Untreated Psychosis	<1 year					
	>1 year	0.381	1.46	0.72	2.96	0.289

**Table 5:** Multiple logistic regression analysis of comorbidity of substance abuse, physical activity, and fluanxol depot with metabolic syndrome in patients with schizophrenia.

Variable	B	Adjusted Odd Ratio	95% CI Low Up		Wald (df)	*p*-Value
Substance	1.20	3.32	1.02	10.81	4.0 (1)	0.047
Physical Activity	−0.88	0.41	0.14	1.21	2.6 (1)	0.107
I.M. Fluanxol	0.76	2.12	0.91	4.94	3.0 (1)	0.082
